# Physical coupling of H3K4me3 demethylases and Polycomb repressive complex 2 to accelerate flowering in rice

**DOI:** 10.1093/plphys/kiae172

**Published:** 2024-03-22

**Authors:** Hua Xuan, Nan Shi, Jianhao Chen, Yili Jiang, Hao Zhang, Chuanliang Chu, Shaoqing Li, Xiangsong Chen, Hongchun Yang

**Affiliations:** State Key Laboratory of Hybrid Rice, College of Life Sciences, Wuhan University, Wuhan 430072, China; Hubei Hongshan Laboratory, Wuhan 430072, China; State Key Laboratory of Hybrid Rice, College of Life Sciences, Wuhan University, Wuhan 430072, China; Hubei Hongshan Laboratory, Wuhan 430072, China; State Key Laboratory of Hybrid Rice, College of Life Sciences, Wuhan University, Wuhan 430072, China; Hubei Hongshan Laboratory, Wuhan 430072, China; State Key Laboratory of Hybrid Rice, College of Life Sciences, Wuhan University, Wuhan 430072, China; Hubei Hongshan Laboratory, Wuhan 430072, China; State Key Laboratory of Hybrid Rice, College of Life Sciences, Wuhan University, Wuhan 430072, China; Hubei Hongshan Laboratory, Wuhan 430072, China; State Key Laboratory of Hybrid Rice, College of Life Sciences, Wuhan University, Wuhan 430072, China; Hubei Hongshan Laboratory, Wuhan 430072, China; State Key Laboratory of Hybrid Rice, College of Life Sciences, Wuhan University, Wuhan 430072, China; Hubei Hongshan Laboratory, Wuhan 430072, China; State Key Laboratory of Hybrid Rice, College of Life Sciences, Wuhan University, Wuhan 430072, China; Hubei Hongshan Laboratory, Wuhan 430072, China; State Key Laboratory of Hybrid Rice, College of Life Sciences, Wuhan University, Wuhan 430072, China; Hubei Hongshan Laboratory, Wuhan 430072, China; RNA Institute, Wuhan University, Wuhan 430072, China

## Abstract

Two H3K4me3 demethylases physically interact with the Polycomb repressive complex 2, thereby altering methylation of a key flowering locus and promoting rice flowering.

Dear Editor,

Flowering time is a crucial breeding trait in rice (*Oryza sativa*), determining planting area and yield (Izawa 2007; Sun et al. 2014). Epigenetic modifications like histone 3 lysine 4 trimethylation (H3K4me3) and H3K27me3 are essential in regulating the expression levels of flowering genes (Wang et al. 2013; Yang et al. 2013; Choi et al. 2014; Xie et al. 2015; Jiang et al. 2018). However, knowledge of their coordinated regulation is limited. Here, we show that H3K4me3 demethylase family proteins JmjC-domain-containing protein 703 (JMJ703) and JMJ704 interact with Polycomb repressive complex 2 (PRC2) core component EMBRYONIC FLOWER 2b (EMF2b). They coordinately reduce H3K4me3 and increase H3K27me3 at *LEAFY COTYLEDON 2 and FUSCA 3-LIKE 1* (*OsLFL1*) and globally, thereby repressing *OsLFL1* expression to promote flowering.

A comprehensive genetic network for flowering time regulation has been established in rice, like the early heading date1 (Ehd1) pathway (Doi et al. 2004; Lee and An 2015). OsLFL1 suppresses *Ehd1* expression under long day conditions (Peng et al. 2007; Itoh et al. 2010). PRC2 complex and its accessorial plant homeodomain protein, rice leaf inclination 2/VIN3-LIKE 2 (OsLC2/VIL2), repress *OsLFL1* expression by depositing H3K27me3 at its chromatin (Wang et al. 2013; Yang et al. 2013; Xie et al. 2015). Exploring *OsLFL1* regulation will improve our understanding of rice floral transition.

To identify factors that cooperate with PRC2, we used EMF2b to screen a library containing 1,588 predicted rice transcription factors by the yeast two-hybrid assay (Y_2_H; Fig. 1A; Shi et al. 2021). Twenty candidate proteins were identified, including JMJ704 (LOC_Os05g23670; Supplementary Table S1), which belongs to the H3K4me3 demethylase KDM5 family. JMJ704 and EMF2b association supplies an opportunity to address the cooperated function of H3K4me3 demethylase with PRC2. We confirmed JMJ704-EMF2b protein interaction and mapped their interaction surface by Y_2_H (Fig. 1, B–D). The interaction was mainly mediated by the N-terminal of JMJ704, containing the JmjN and JmjC domains and the VRN2-EMF2-FIS2-SU(Z)12 (VEFS) box of EMF2b (Fig. 1, C and D). The interaction was further confirmed by split firefly luciferase complementation (SFLC) assay in *Nicotiana benthamiana* leaves and co-immunoprecipitation (Co-IP) in rice protoplast (Fig. 1, E and F).

**Figure 1.**
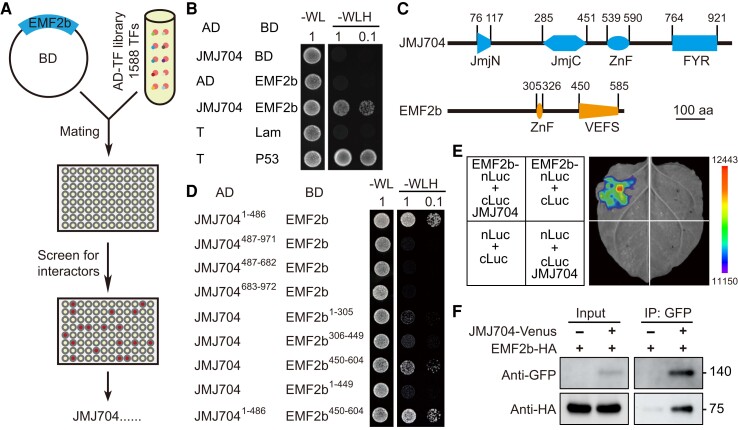
PRC2 associates with H3K4 demethylase JMJ704. **A)** The scheme of mating-based yeast two-hybrid (Y_2_H) screening. EMF2b bait yeast strain was mated with a rice TFs library containing 1,588 TFs in 96-well plates. After mating, the cultures were plated on selective solid medium plates. The positive clones like JMJ704 were pricked out. AD, activation domain; BD, binding domain; TF, transcription factor. **B)** JMJ704 interacts with EMF2b in yeast cells. T, SV40 large T-antigen; Lam, Lamin C. **C)** Schematic illustrations of the JMJ704 and EMF2b domains. The numbers indicate the positions of the amino acid residues in the constructs used for Y_2_H. ZnF, Zinc finger; FYR, FY-rich; VEFS, VRN2-EMF2-FIS2-SU(Z)12; aa, amino acid. **D)** Rough mapping of JMJ704 and EMF2b interaction domains. The fusion proteins or truncated proteins were constructed as shown in C). In B and D), the pairs of indicated plasmids were co-transformed and grown on selective solid media lacking tryptophane, leucine, and histidine (SD-WLH). Representative pictures from three independent experiments are shown. **E)** SFLC assay shows the physical association of JMJ704 with EMF2b in *Nicotiana benthamiana*. The pairs of co-infiltrated plasmids are presented in each quadrant. Images are representative of three independent experiments. **F)** EMF2b-HA is co-immunoprecipitated with JMJ704-Venus in rice protoplasts. Hemagglutinin (HA)-tagged EMF2b (EMF2b-HA) and Venus-tagged JMJ704 (JMJ704-Venus), or EMF2b-HA alone served as a negative control, were expressed. Proteins were extracted and subjected to Co-IP. Samples before and after immunoprecipitation were detected by anti-HA and anti-GFP. Images are representative of two independent experiments.

We generated two *JMJ704* mutants (*jmj704-3* and *jmj704-4*), and no apparent developmental defect was observed (Supplementary Fig. S1). JMJ703 (LOC_Os05g10770) is the closest homolog of JMJ704 (Lu et al. 2008; Chen et al. 2013; Song et al. 2018). They had similar expression patterns in different tissues, showing higher expression levels in the leaf and the flag leaf (Supplementary Fig. S2). Like JMJ704, JMJ703 interacted with EMF2b in SFLC and Co-IP assays (Fig. 2, A and B). We obtained two loss-of-function mutants of *JMJ703* (*jmj703-5* and *jmj703-6*), and did not detect any other obvious phenotype except for the reported semi-dwarf plants (Supplementary Fig. S3; Chen et al. 2013). JMJ703 interacted with JMJ704 in *Nicotiana benthamiana* leaves and rice protoplast (Supplementary Fig. S4, A and B). Transiently expressing JMJ703 or JMJ704 in the protoplasts of *35S::EMF2b-Flag-HA/*Nip transgenic plants, both interacted with EMF2b-Flag-HA detected by the Co-IP assays (Supplementary Fig. S4C). Thus, we generated two double mutants (*jmj703 jmj704-1* and *jmj703 jmj704-2*) in which the mutations caused truncations of JMJ703 and JMJ704 (Supplementary Fig. S5). Besides semi-dwarf plants, both mutants displayed a late flowering phenotype (Fig. 2, C and D). Thus, JMJ703 and JMJ704 redundantly accelerate flowering in rice.

**Figure 2.**
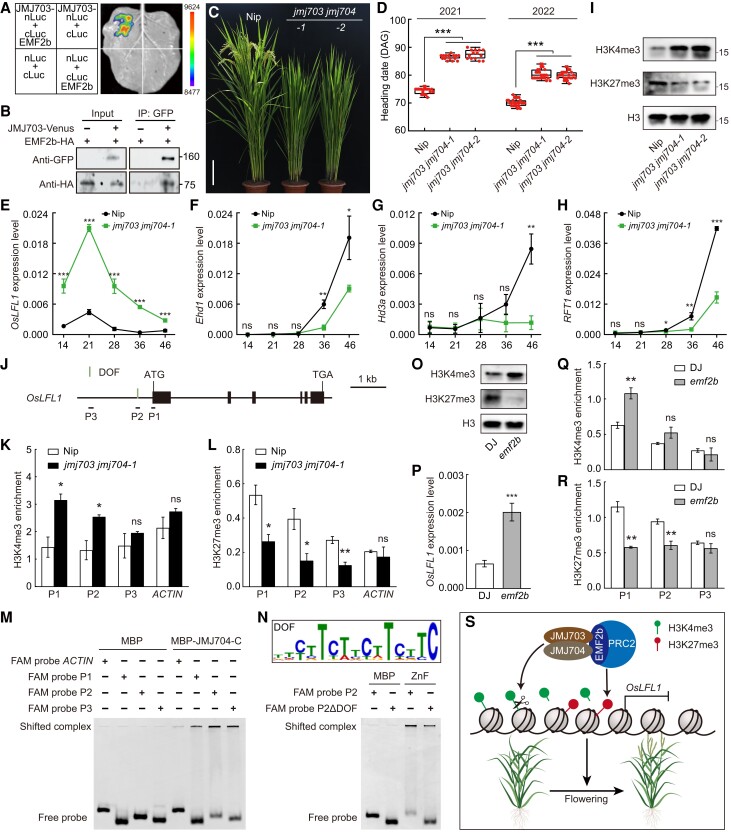
H3K4 demethylases coordinate with PRC2 to promote flowering in rice. **A)** SFLC assay shows the physical association of JMJ703 with EMF2b in *Nicotiana benthamiana*. The pairs of co-infiltrated plasmids are presented in each quadrant. Images are representative of three independent experiments. **B)** EMF2b-HA is co-immunoprecipitated with JMJ703-Venus in rice protoplasts. EMF2b-HA and JMJ703-Venus, or EMF2b-HA alone served as a negative control, were expressed. Proteins were extracted and subjected to Co-IP. Samples before and after immunoprecipitation were detected by anti-HA and anti-GFP. Images are representative of two independent experiments. **C)** Representative images of wild-type Nipponbare (Nip) and the *jmj703 jmj704* double mutants at the heading stage grown in the paddy fields at Wuhan in the summer. Scale bar, 15 cm. **D)** Boxplot showing heading date of Nip and the *jmj703 jmj704* double mutants grown in the paddy fields at Wuhan in the summer. The lower and upper ±1.5 quartiles are indicated by whiskers, the lower and upper ends of the boxes indicate the 25th and 75th quartiles, and the line across the middle of the box identifies the median sample value (*n* ≥ 14) (***P < 0.001; unpaired two-tailed *t*-tests). DAG, days after germination. **E–H)** Relative expression level of *OsLFL1***E**), *Ehd1***F**), *Hd3a***G**), and *RFT1***H**) in Nip and the *jmj703 jmj704*. RNA was isolated from the second leaf blades collected from the top of the indicated plants. Values are mean ± s.d. (*n* = 3 biological replicates). The *OsACTIN1* gene was used as an internal reference control (*P < 0.05; **P < 0.01; ***P < 0.001; ns, no significant, *P* ≥ 0.05; unpaired two-tailed *t*-tests). **I)** The H3K4me3 and H3K27me3 levels in indicated genotypes. Protein was extracted and detected by corresponding antibodies showing on the left. The H3 level was used as the loading control. **J)** Genomic structure of *OsLFL1* and regions tested in chromatin immunoprecipitation (ChIP) and EMSA assays. Green vertical line indicates DNA-binding with one finger (DOF) motif. P1, probe 1; P2, probe 2; P3, probe 3; DOF, DNA binding with one finger. **K** and **L**) Levels of H3K4me3 **K**) and H3K27me3 **L**) at the *OsLFL1* chromatin assayed by ChIP-qPCR. Values are mean ± SEM (*n* = 3 biological replicates). Each examined region is shown below locus in J). (*P < 0.05; **P < 0.01; ns, no significant, *P* ≥ 0.05; unpaired two-tailed *t*-tests). **M)** EMSA testing JMJ704 binding to *OsLFL1* DNA fragments. The purified MBP-JMJ704-C (487–971; 2 *μ*g) were incubated with 10 nmol carboxyfluorescein (FAM)-labeled probes. Probe *ACTIN* was used as a negative control. **N)** The ZnF domain of JMJ704 binds to *OsLFL1* P2 fragment through the DOF motif. The purified MBP-JMJ704-ZnF (487–682; 2 *μ*g) were incubated with 10 nmol FAM-labeled probes. **O)** Comparison of the H3K4me3 and H3K27me3 levels between wild-type DJ and *emf2b* plants. Protein was extracted and detected by corresponding antibodies showing on the left. The H3 level was used as the loading control. **P)** Relative expression level of *OsLFL1* in DJ and *emf2b*. Values are mean ± s.d. (*n* = 4 biological replicates). The *OsACTIN1* gene was used as an internal reference control (***P < 0.001; unpaired two-tailed *t*-tests). **Q** and **R**) Levels of H3K4me3 **Q)** and H3K27me3 **R)** at the *OsLFL1* chromatin assayed by ChIP-qPCR. Values are mean ± SEM (*n* = 3 biological replicates). Each examined region is shown below locus in J) (**P < 0.01; ns, no significant, *P* ≥ 0.05; unpaired two-tailed *t*-tests). **S)** Model for regulatory network controlling flowering time by JMJ703/JMJ704-PRC2 in rice.

LC2-PRC2 complex binds to the promoter of *OsLFL1*, thereby catalyzing H3K27me3 to repress its expression (Wang et al. 2013; Yang et al. 2013; Xie et al. 2015). We measured the expression levels of *OsLFL1* and its downstream genes *Ehd1*, *Heading date 3a* (*Hd3a*), and *RICE FLOWERING TIME LOCUS T1* (*RFT1*) in a series of developmental times. *OsLFL1* was continuously up-regulated (Fig. 2E); *Ehd1*, *Hd3a*, and *RFT1* displayed lower expression in *jmj703 jmj704-1* before flowering (Fig. 2, F–H). The global level of H3K4me3 was increased in *jmj703 jmj704*, and H3K27me3 level was decreased (Fig. 2I), supporting that H3K4me3 inhibits PRC2 activity (Pasini et al. 2008; Schmitges et al. 2011). We also detected increased H3K4me3 and reduced H3K27me3 at the *OsLFL1* locus in *jmj703 jmj704-1* plants (Fig. 2, J–L), suggesting H3K4me3 demethylation potentially promotes H3K27me3 deposition at *OsLFL1*. Previous studies show that zinc finger (ZnF) domains can bind DNA in plants (Ciftci-Yilmaz and Mittler 2008; Cui et al. 2016). We purified the Maltose binding protein (MBP)-tagged C-terminal of JMJ704 (MBP-JMJ704-C) containing ZnF and FY-rich (Phenylalanine/tyrosine-rich, FYR) domains, and found that it could bind to these probes from *OsLFL1* (P1, P2, and P3) but not the probe of *OsACTIN1* (LOC_Os03g50885), which is not likely to be a target for PRC2 and H3K4me3 demethylases, detected by electrophoretic mobility shift assay (EMSA; Fig. 2, J and M, and Supplementary Fig. S6A). MBP-JMJ704-C had relatively higher affinity against P2 (Fig. 2M). The binding activity at P1 and P2 of *OsLFL1* was disrupted by deleting the ZnF domain (MBP-JMJ704-C ΔZnF; Supplementary Fig. S6A). The ZnF domain alone could bind to these two probes with slightly reduced levels (Supplementary Fig. S6A). These data support that JMJ704 binds to *OsLFL1* DNA mainly through the ZnF domain in vitro. JMJ703 also binds to *OsLFL1* DNA, and the binding activity is also mainly dependent on ZnF domain (Supplementary Fig. S6B). JMJ704/JMJ703 and AtJMJ14 belong to KDM5/JARID1 group proteins with a conserved C5HC2-type ZnF domain (Supplementary Fig. S6C). A previous study suggested that the DNA motif DOF (DNA-binding with one finger) is one of the enriched motifs in the binding regions of AtJMJ14 (Fig. 2N; Wang et al. 2023). We only found the DOF motif in P2, and the lack of the DOF motif (P2ΔDOF) reduced the binding level of JMJ704-ZnF (Fig. 2, J and N). Our data suggest that JMJ703/JMJ704 bind to *OsLFL1*, reducing H3K4me3 and promoting H3K27me3 levels at its chromatin, thereby promoting *OsLFL1* in flowering time regulation.

As H3K4me3 inhibits PRC2 activity, understanding how H3K4me3 is coordinately removed during PRC2 silencing is critical. To address the cooperated function of JMJ703/JMJ704 and PRC2, we got an *emf2b* mutant in Dongjin (DJ) background (Xie et al. 2015). Mutation of *EMF2b* resulted in reduced H3K27me3 and increased H3K4me3 globally (Fig. 2O). Like *jmj703 jmj704*, *emf2b* plants displayed higher expression of *OsLFL1*; increased H3K4me3 and decreased H3K27me3 at the *OsLFL1* chromatin (Fig. 2, P–R). These suggest that JMJ703/JMJ704-PRC2 is functionally coupled in repressing *OsLFL1*. Our data show that JMJ703 and JMJ704 physically associate with PRC2 to reduce H3K4me3 and increase H3K27me3 globally and at the *OsLFL1* locus to control flowering time (Fig. 2S).

## Supplementary Material

kiae172_Supplementary_Data

## Data Availability

The data underlying this article will be shared on reasonable request to the corresponding author.

## Supplementary data

The following materials are available in the online version of this article.

**Supplementary Figure S1**. *jmj704* mutant plants have no obvious developmental defect.

**Supplementary Figure S2**. *JMJ704* and *JMJ703* expression levels in various organs revealed by RT-qPCR.

**Supplementary Figure S3**. Heading time of *jmj703* plants.

**Supplementary Figure S4**. The protein interactions among JMJ704, JMJ703, and EMF2b.

**Supplementary Figure S5**. Targeted mutagenesis of *JMJ703 JMJ704* double mutant by CRISPR/Cas9 technology.

**Supplementary Figure S6**. JMJ704/JMJ703 bind to *OsLFL1* promoter in vitro.

**Supplementary Table S1**. List of the candidate transcription factors identified by yeast two-hybrid screening.

**Supplementary Table S2**. List of the primers used in this study.

## Dive Curated Terms

The following phenotypic, genotypic, and functional terms are of significance to the work described in this paper:

protein CHEBI: CHEBI:36080

proteins CHEBI: CHEBI:36080

KDM5 Gramene: KDM5 (lysine-specific demethylase 5)
KDM5 Araport: KDM5 (lysine-specific demethylase 5)

PHD Gramene: PHD (plant homeodomain)
PHD Araport: PHD (plant homeodomain)

probe CHEBI: CHEBI:50406
