# Supplementary material for: Physical coupling of H3K4me3 demethylases and Polycomb repressive complex 2 to accelerate flowering in rice
Source: Plant Physiol. 2024 Mar 22;195(3):1802–6. doi: 10.1093/plphys/kiae172 (PMC11213247; doi:10.1093/plphys/kiae172)
Supplement: kiae172_Supplementary_Data [file kiae172_supplementary_data.zip › Supplemental Data.pdf]

Supplemental Materials and Methods

Plant materials and growth conditions

Rice (*Oryza sativa subsp. japonica*) variety Nipponbare (Nip) and Dongjin (DJ) were used in this study. All the plants were grown either in the greenhouse under 14 hours light and 10 hours dark at 28°C or in the paddy field of the Wuhan area (30.6, 114.5) in summer.

To generate mutants, two small guide RNAs (sgRNAs) targeting *JMJ703* (LOC_Os05g10770) and *JMJ704* (LOC_Os05g23670) were designed, respectively (Xie et al., 2017); the mutants were generated by CRISPR/Cas9 technology in Nip (Miao et al., 2013). All the mutants were identified and confirmed by Sanger sequencing. The homozygous mutants without CRISPR/Cas9 transgene were obtained and used in the characterizations. Primers used for generating CRISPR-Cas9 vector were listed in Supplemental Table S2. *emf2b* was described previously (Xie et al., 2015).

To generate *35S::EMF2b-Flag-HA*/Nip stable transgenic plants, the coding sequence (CDS) of *EMF2b* was fused with *Flag-HA* driven by a Cauliflower mosaic virus (CaMV) *35S* promoter in a modified pCAMBIA1300 vector. The plasmid was introduced into Nip by the Agrobacterium-mediated transformation. The transgene homozygous lines were selected according to resistance selection. All primers used were listed in Supplemental Table S2.

Yeast two-hybrid assay

For the yeast two-hybrid screen, the CDS of *EMF2b* was amplified and cloned into a pGBKT7 vector (Clontech), then transformed into the yeast strain Y_2H Gold. The EMF2b was used as the bait to screen a library containing 1,588 predicted transcription factors of rice constructed in pGADT7 by OE Biotech (Shanghai, China) (Shi et al., 2021). *EMF2b*, *JMJ704*, and all the truncations were amplified and cloned into the Y_2H vectors pGADT7 or pGBKT7. The experiments were done according to the Yeast

Protocols Handbook. Medium lacking tryptophane, leucine, and histidine (SD-WLH) was used for selection. 5 µl aliquots of yeast cell suspensions (OD_{600} = 1 and 0.1) were applied for spotting on the plates. All the primers used in cloning are listed in Supplemental Table S2.

Split firefly luciferase complementation (SFLC) assay

The SFLC assay was performed as described previously (Chen et al., 2008; Zhang et al., 2022). Full-length CDS of *EMF2b*, *JMJ703*, and *JMJ704* were amplified and inserted into the pCAMBIA1300-35S-nLuc or pCAMBIA1300-35S-cLuc vectors. The pairs of constructs were co-infiltrated into *Nicotiana benthamiana* leaves respectively. Images were photographed by NightSHADE LB 985 (Berthold Technologies). Each assay was repeated at least two times, similar results were obtained. All the primers used in cloning are listed in Supplemental Table S2.

Co-immunoprecipitation (Co-IP) and western blotting

Co-IP experiments were performed in rice protoplasts. The protoplasts were transfected with 100 µg of each target plasmid (all the primers used in cloning are listed in Supplemental Table S2) and then incubated overnight at 28°C in the dark. The cultured cells were harvested and lysed by IP buffer (50 mM Tris-HCl pH 7.5, 150 mM NaCl, 1 mM EDTA, 1% Triton X-100, 0.1% SDS, 5 mM DTT, 1 mM PMSF and Protease inhibitor cocktail). After clearing by centrifugation, the supernatants were incubated with anti-GFP beads (ChromoTek) at 4°C for 3 hours. The beads were washed 5 times with IP buffer. The protein complex was eluted by 2× loading buffer (100 mM Tris-HCl pH 6.8, 4% SDS, 20% glycerol, 0.004% bromophenol blue, and 2% β-mercaptoethanol). To detect H3K4me3 and H3K27me3 levels, nuclei were isolated from the second leaf blades collected from the top of three-week-old plants and nuclear proteins were extracted as described previously (Yang et al. 2017). Immunoprecipitates or nuclear proteins were separated by SDS-PAGE and transferred to a nitrocellulose membrane (GE Healthcare, 10600001). Antibodies of anti-HA (ABclonal, AE008), anti-GFP

(Roche, 11814460001), anti-H3K27me3 (ABclonal, A2363), anti-H3K4me3 (Abclonal, A2357), and anti-H3 (Abclonal, A2348) were used to detect target proteins. The images were photographed by GE Amersham Imager AI680.

RNA expression analysis and RT-qPCR

Total RNA was extracted from indicated plant materials using the hot phenol method according to the previous description (Zhang et al., 2022; Yang et al., 2017). Genomic DNA contamination was cleaned by DNase I (Roche, 04716728001) following the manufacturer's guidelines. The full-length cDNA was synthesized with 2 μg total RNA by the HiScript® II 1st Strand cDNA Synthesis Kit (Vazyme, R211-01) with Oligo dT. The quantitative real-time PCR was performed with the ChamQ SYBR® qPCR Master Mix (Vazyme, Q311-02) on a LightCycler 480 instrument (Roche). The *OsACTIN1* (LOC_Os03g50885) was used as an internal reference. All primers are listed in Supplemental Table S2.

Chromatin immunoprecipitation (ChIP) and qPCR assay

The ChIP assay was performed as described previously with modifications (Yang et al., 2017). For Nip and the *jmj703 jmj704*, 2 g of the second leaf blades collected from the top of three-week-old plants were used. For DJ and *emf2b*, 2 g of the seedling from the two-week-old plants were used. Immunoprecipitations were performed with anti-H3 (Abclonal, A2348), anti-H3K4me3 (Abclonal, A2357), and anti-H3K27me3 (ABclonal, A2363). The immunoprecipitated DNA fragments were then purified after reverse crosslink and amplified by real-time quantitative PCR. *OsACTIN1* was used as the internal control for each sample. Data are represented as the ratio to H3. Relevant primers are listed in Supplemental Table S2.

Protein expression and purification from *Escherichia coli*

The truncations of *JMJ703* and *JMJ704* were amplified and cloned into the MBP vectors, then transformed into *Escherichia coli* BL21 (DE3) strain. Pre-cultured cells

containing responding plasmids were grown at 37°C to reach OD_{600} about 0.6. Protein expression was induced by the addition of 0.5 mM isopropyl β-D-1-thiogalactopyranoside (IPTG). After being grown for 16 hours at 16°C, cells were harvested and resuspended in suspension buffer (10 mM HEPES-KOH pH 7.5, 150 mM NaCl, and 1 mM PMSF). The cells were lysed using a high-pressure homogenizer (ATS Engineering) and centrifuged at 16,000 g for 1 hour. The supernatants flowed through a Dextrin beads 6FF column (Smart-lifesciences, SA026100). The Dextrin beads were washed with wash buffer (10 mM HEPES-KOH pH 7.5, 150 mM NaCl, and 1 mM PMSF), proteins were eluted by elution buffer (10 mM HEPES-KOH pH 7.5, 150 mM NaCl, 10 mM Maltose, and Protease inhibitor cocktail). Proteins were concentrated by the Amicon® Mltra filter device (Millipore, UFC501008). Relevant primers are listed in Supplemental Table S2.

Electrophoretic Mobility Shift Assay (EMSA)

The EMSA assay was conducted with LightShift Chemiluminescent RNA EMSA Kit (Thermo, 89880) according to the protocol described. The 5'-FAM-labeled probes (10 nM) were incubated with purified proteins (2 μg) at 25°C for 30 min. Then samples were loaded to electrophoresis with 0.5 × TBE buffer at 4°C for 3 h. Labeled probes were visualized by chemiluminescence apparatus (GE Amersham Imager AI680).

Statistical analysis

Statistical analysis was carried out by GraphPad Prism 8.0 software as stated in the figure legends. The sample size of each experiment was indicated in the figure legends.

References

Chen H, Zou Y, Shang Y, Lin H, Wang Y, Cai R, Tang X, Zhou JM. Firefly luciferase complementation imaging assay for protein-protein interactions in plants. Plant Physiol. 2008:**146**(2):368-376.

Miao J, Guo D, Zhang J, Huang Q, Qin G, Zhang X, Wan J, Gu H, Qu LJ. Targeted mutagenesis in rice using CRISPR-Cas system. Cell Res. 2013:**23**(10):1233-1236.

Shi J, Zhao B, Zheng S, Zhang X, Wang X, Dong W, Xie Q, Wang G, Xiao Y, Chen F et al. A phosphate starvation response-centered network regulates mycorrhizal symbiosis. Cell. 2021:**184**(22):5527-5540.

Xie S, Chen M, Pei R, Ouyang Y, Yao J. *OsEMF2b* acts as a regulator of flowering transition and floral organ identity by mediating H3K27me3 deposition at *OsLFL1* and *OsMADS4* in rice. Plant Mol Biol Rep. 2015:**33**(1):121-132.

Xie X, Ma X, Zhu Q, Zeng D, Li G, Liu YG. CRISPR-GE: A Convenient Software Toolkit for CRISPR-Based Genome Editing. Mol Plant. 2017:**10**(9):1246-1249.

Yang H, Berry S, Olsson TSG, Hartley M, Howard M, Dean C. Distinct phases of Polycomb silencing to hold epigenetic memory of cold in *Arabidopsis*. Science. 2017:**357**(6356):1142-1145.

Zhang X, Li W, Liu Y, Li Y, Li Y, Yang W, Chen X, Pi L, Yang H. Replication protein RPA2A regulates floral transition by cooperating with PRC2 in *Arabidopsis*. New Phytol. 2022:**235**(6):2439-2453.

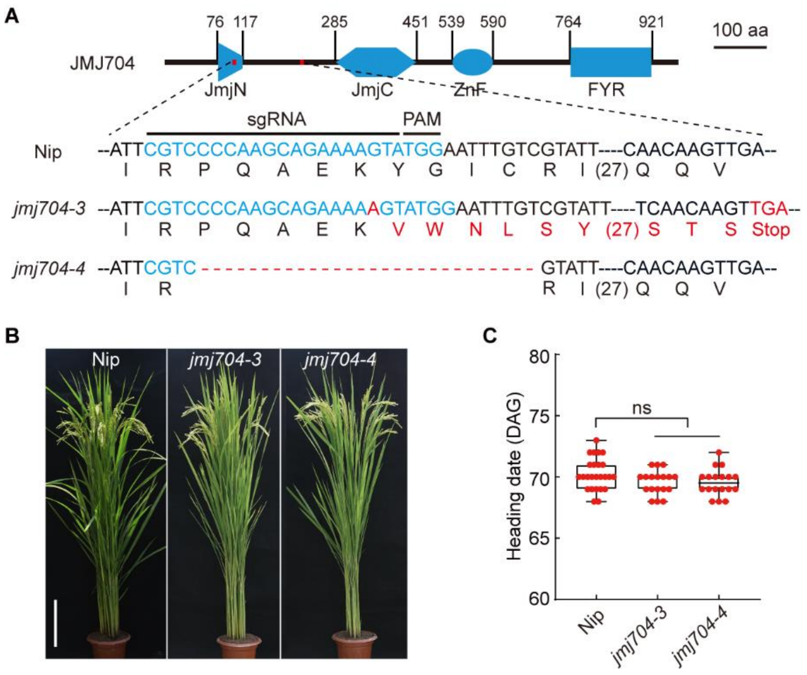

Supplemental Figure S1. *jmj704* mutant plants have no obvious developmental defect. **A)** Targeted mutagenesis of *JMJ704* gene by CRISPR/Cas9 technology. A schematic picture shows the conserved domains of JMJ704. The position of the selected small guide RNA (sgRNA) and corresponding protospacer adjacent motif (PAM) for each target site are displayed. The mutant alleles are aligned to the reference genome sequence. The indels are shown in red letters or dashes. Nip, Nipponbare; ZnF, Zinc finger; FYR, FY-rich; aa, amino acid. **B)** Representative images of Nip and the *jmj704* plants at heading stage grown in the paddy fields at Wuhan, in the summer. Scale bar, 15 cm. **C)** Boxplot showing heading date of Nip and the *jmj704* plants grown in the paddy fields at Wuhan, in the summer. The lower and upper ± 1.5 quartiles are indicated by whiskers, the lower and upper ends of the boxes indicate the 25th and 75th quartiles, and the line across the middle of the box identifies the median sample value ($n \geq 18$) (ns, no significant, $P \geq 0.05$; unpaired two-tailed t-tests). DAG, Days after germination.

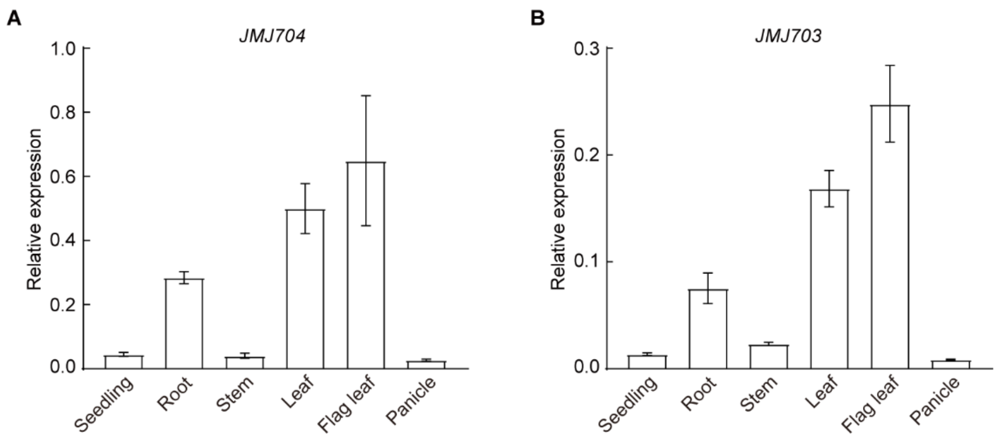

Supplemental Figure S2. *JMJ704* **A)** and *JMJ703* **B)** expression levels in various organs revealed by RT-qPCR. Values are mean ± s.d. (n = 3 biological replicates). The *OsACTIN1* gene was used as an internal reference control.

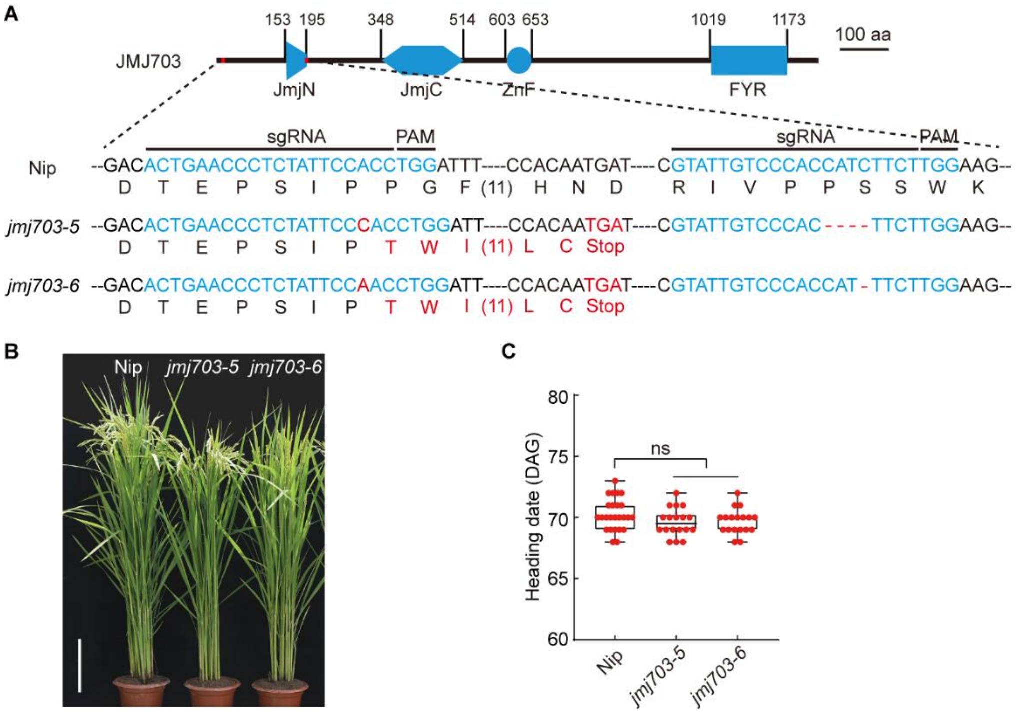

Supplemental Figure S3. Heading time of *jmj703* plants. **A)** Targeted mutagenesis of *JMJ703* gene by CRISPR/Cas9 technology. A schematic picture shows the conserved domains of JMJ703. The position of the selected sgRNA and corresponding PAM for each target site are displayed. The mutant alleles are aligned to the reference genome sequence. The indels are shown in red letters or dashes. **B)** Representative images of Nip and the *jmj703* at heading stage grown in the paddy fields at Wuhan, in the summer. Scale bar, 15 cm. **C)** Boxplot showing heading date of Nip and the *jmj703* plants grown in the paddy fields at Wuhan, in the summer. The lower and upper ± 1.5 quartiles are indicated by whiskers, the lower and upper ends of the boxes indicate the 25th and 75th quartiles, and the line across the middle of the box identifies the median sample value (n ≥ 18) (ns, no significant, P ≥ 0.05; unpaired two-tailed t-tests).

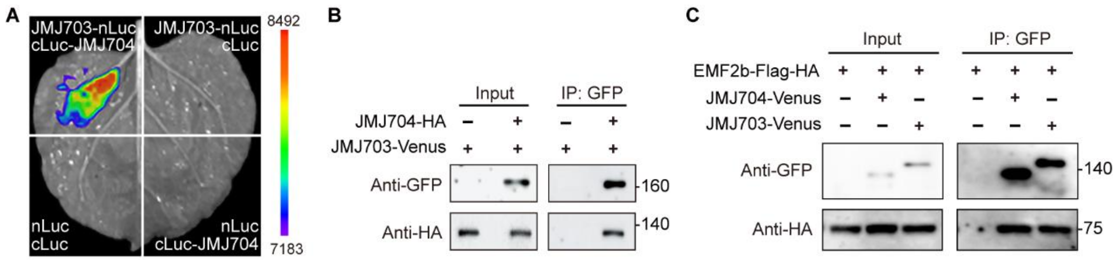

Supplemental Figure S4. The protein interactions among JMJ704, JMJ703, and EMF2b. **A)** Split firefly luciferase complementation (SFLC) assay shows the interaction of JMJ703 with JMJ704 in *Nicotiana benthamiana*. The pairs of co-infiltrated plasmids are presented in each quadrant. Images are representative of three independent experiments. **B)** JMJ704-HA is co-immunoprecipitated with JMJ703-Venus in rice protoplasts. Hemagglutinin (HA)-tagged JMJ704 (JMJ704-HA) and Venus-tagged JMJ703 (JMJ703-Venus), or JMJ704-HA alone served as a negative control, were expressed. Proteins were extracted and subjected to co-immunoprecipitation. Samples before and after immunoprecipitation were detected by anti-HA and anti-GFP. Images are representative of two independent experiments. **C)** EMF2b is co-precipitated with JMJ703-Venus or JMJ704-Venus. JMJ703-Venus or JMJ704-Venus is transformed into the protoplasts of *35S::EMF2b-Flag-HA*/Nip for co-immunoprecipitation. Non-transformed protoplasts are used as a negative control. Proteins were extracted and subjected to co-immunoprecipitation. Samples before and after immunoprecipitation were detected by anti-HA and anti-GFP. Images are representative of two independent experiments.

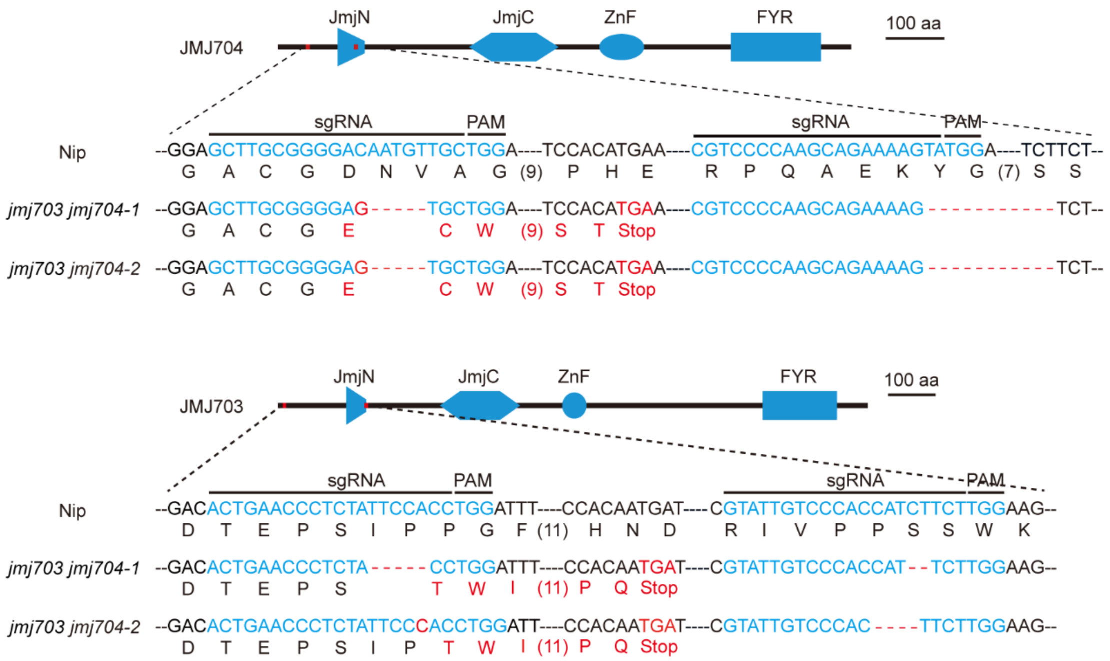

Supplemental Figure S5. Targeted mutagenesis of *JMJ703 JMJ704* double mutants by CRISPR/Cas9 technology. A schematic picture shows the conserved domains of JMJ703 and JMJ704. The position of the selected sgRNA and PAM for each target site are displayed. The mutant alleles are aligned to the reference genome sequence. The indels are shown in red letters or dashes.

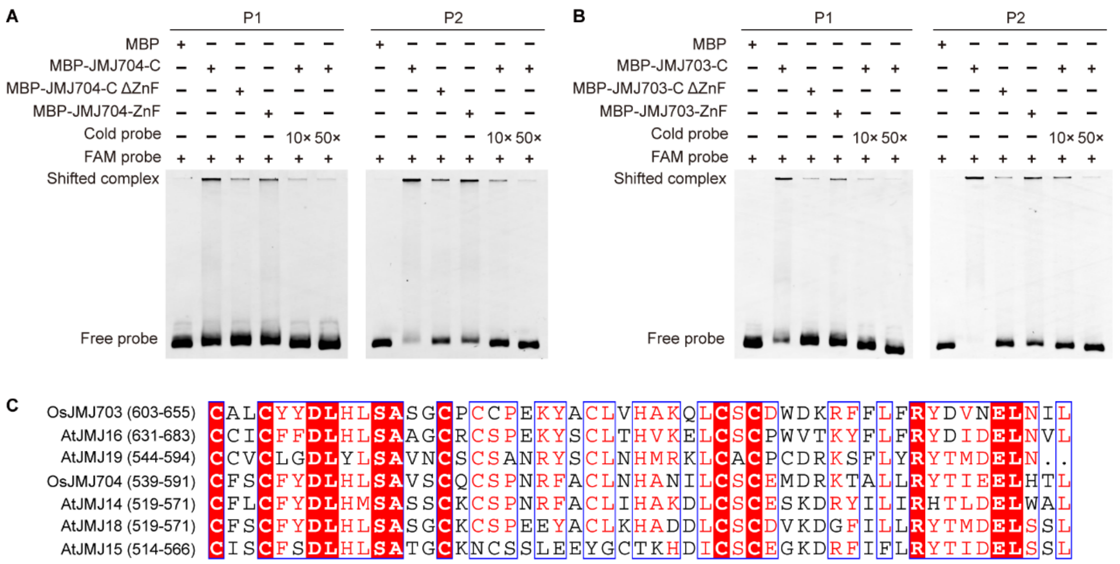

Supplemental Figure S6. JMJ704/JMJ703 bind to *OsLFL1* promoter in vitro. **A** and **B)** EMSA analysis showing JMJ704 **A)** and JMJ703 **B)** binding to *OsLFL1* DNA fragments. The purified MBP-JMJ703/JMJ704 truncation proteins (2 μg) were incubated with 10 nmol FAM-labeled probes. For the competition test, non-labeled probes with different concentrations (10 or 50 times) were added. Each examined region is shown in **Fig 2J**. JMJ704-C (487-971), JMJ704-C ΔZnF (487-529, 601-971), and JMJ704-ZnF (487-682). JMJ703-C (561-1138), JMJ703-C ΔZnF (561-594, 661-1138), and JMJ703-ZnF (561-700). MBP, Maltose binding protein; P1, Probe 1; P2, Probe 2; ZnF, Zinc finger; FAM, Carboxyfluorescein. **C)** Alignment of the zinc finger (ZnF) domains of KDM5/JARID1 group proteins in *Arabidopsis* and rice. Amino acid residues with red backgrounds represent that they are completely consistent in all species. Red amino acid residues represent that they are highly consistent in all species. Among them, the consistent amino acid residues in the blue box are greater than or equal to 50%. The numbers in parentheses represent the amino acid positions of the ZnF domain in these proteins.
